# ﻿*Ardisiawhitmorei* (Primulaceae-Myrsinoideae), a new species from north east of Peninsular Malaysia

**DOI:** 10.3897/phytokeys.204.86647

**Published:** 2022-08-04

**Authors:** Avelinah Julius, Timothy M.A. Utteridge

**Affiliations:** 1 Forest Research Institute Malaysia, Kepong, Selangor, 52109, Malaysia Forest Research Institute Malaysia Kepong Malaysia; 2 Royal Botanic Gardens, Kew, Richmond, TW9 3AE, UK Royal Botanic Gardens Richmond United Kingdom

**Keywords:** Endemic, Ericales, Gunung Padang, IUCN status, Malesia, *Stylardisia*, taxonomy, Terengganu

## Abstract

*Ardisiawhitmorei* Julius & Utteridge, **sp. nov.** (Primulaceae-Myrsinoideae), a member of ArdisiasubgenusStylardisia on account of the style protruding from the closed petals prior to anthesis, is herein described and illustrated as a new species. This new species is easily distinguished by the combination of the inflorescences with a slender rachis branched to two orders, the corolla lobes are abaxially glabrous with usually up to only two gland-dots near the apex and the brochidrodromous secondary veins with double loops near the margin.

## ﻿Introduction

The genus *Ardisia* Sw. is one of the largest tropical genera in the Primulaceae subfamily Myrsinoideae (containing the woodier, tropical members), having a pantropical distribution with approximately 725 species (POWO 2022). In Peninsular Malaysia, the last comprehensive account of the genus was that of Stone (1989) who treated 74 species in an annotated key in the Tree Flora of Malaya (because most *Ardisia* species do not reach the arborescent limit to merit full descriptions in the Tree Flora). An additional five species were added by Hu (2002; two species), Julius and Utteridge (2012, 2021; two species) and Julius et al. (2017; one species), bringing the total number of species in Peninsular Malaysia to 79.

*Ardisia* is classified into 16 subgenera (indicated here with the silcrow: §) using characters of habit, leaf morphology, inflorescence position and floral morphology (Mez 1902; Stone 1993; Larsen and Hu 1995), with ten subgenera present in Malesia (see Stone 1982; Larsen and Hu 1995). In Peninsular Malaysia, all these subgenera are present with most speciose groups being §*Tinus* and §*Stylardisia*, with 16 and 15 species, respectively.

A new species from southern Peninsular Malaysia, *Ardisiagasingoides* Julius & Utteridge, was initially assigned to §*Stylardisia*, based on collections of fruiting material (Julius et al. 2017). However, recent molecular results suggest it is better placed in §*Acrardisia* (Julius 2019; Julius et al. 2021). This example shows the importance of having flowering specimens, or sequenced material, available for a definitive subgeneric placement in *Ardisia*. Stone (1989) annotated specimens of §*Stylardisia* in the Herbarium of the Herbarium of the Forest Research Institute Malaysia at Kepong (KEP) during his work for the Tree Flora account, but several could not be identified due to incomplete material. A single fruiting collection from Gunung (G.) Padang collected by Timothy Whitmore in 1969 was annotated by Stone (6 Oct 1980) as ‘*Ardisia* sp. “Y” near *A.sessilis* Scheff. but distinct’, but Stone (1989) did not list this taxon in his annotated key to the genus in his Tree Flora account. Recently, flowering material of the species was collected during an expedition to G. Padang in 2010 (Ummul-Nazrah et al. 2011), allowing us to critically scrutinise the floral and fruit morphology against the known species in the subgenus from Peninsular Malaysia. After careful examination of the specimens and the relevant literature of species known from §*Stylardisia*, we confirm that this is an undescribed taxon and it is formally described and illustrated as new to science here. The new taxon described here brings the number of §*Stylardisia* species native to Peninsular Malaysia to 16.

## ﻿Materials and methods

This study was based on examination of herbarium specimens at K, KEP and the relevant taxonomic literature (e.g. Stone 1982, 1992; Larsen and Hu 1991; Chen and Pipoly 1996; Hu 2002); in addition, specimen images from Global Plants JSTOR (http://plants.jstor.org/), Kew Herbarium Catalogue (http://apps.kew.org/herbcat/gotoHomePage.do) and Plants of the World Online (**POWO**: http://www.plantsoftheworldonline.org/) were consulted. All measurements were taken from herbarium specimens and rehydrated material for the floral description; shape terminology follows Systematics Association Committee (1962). Flowering and fruiting materials are indicated by ‘fl.’and ‘fr.’, respectively. The conservation assessment of the species was undertaken using IUCN categories of threat (see IUCN 2012 and IUCN Standards and Petitions Committee 2022) following the guidelines and procedures developed at FRIM for the Malaysia Plant Red List (Chua and Saw 2006).

## ﻿Taxonomy

### ﻿ Ardisia whitmorei

Taxon classificationPlantaeEricalesPrimulaceae

Julius & Utteridge, sp. nov. (§Stylardisia)

7A38F1AF-338B-5EAE-BE17-7FE4DD689BD9

urn:lsid:ipni.org:names:77302868-1

Fig. 1

#### Diagnosis.

**Figure 1.**
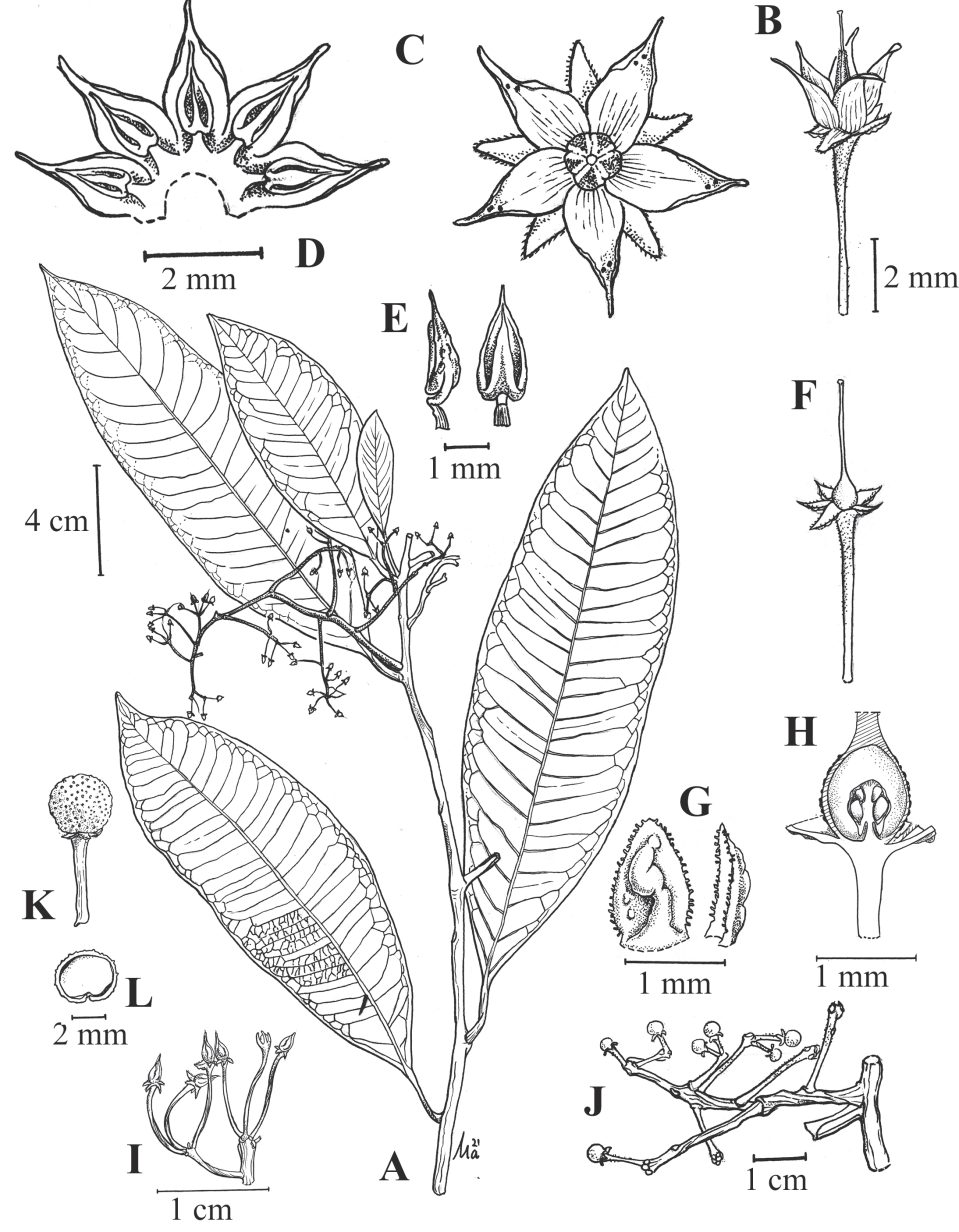
*Ardisiawhitmorei* Julius & Utteridge, sp. nov. **A** flowering leafy twig **B** mature flower **C** aerial view of opened flower **D** flower dissected to show the stamen arrangement **E** anther, lateral (left) and front view (right) **F** petals removed to show calyx and pistil **G** calyx, abaxial (left) and lateral view (right) **H** ovary dissected to show the ovules **I** flower prior to anthesis, showing one flower with exerted style **J** infructescence **K** fruit **L** fruit, cross-section. (Illustration by Mohd Aidil Nordin **A–I** from *Mohd. Hairul et al*., *FRI70884***J–L** from *T.C. Whitmore*, *FRI12727*: scale bar for **C** similar to **D, F** similar to **B** and **K** similar to **L**).

Amongst the Peninsular Malaysian members of subgenus (§) *Stylardisia*, the new species is easily recognised by the following combination characters: lateral veins brochidrodromous with double loops towards the margin and prominent on both surfaces, the relatively large leaves (15–23 cm long), the inflorescences with a slender rachis and branched to two orders and the glabrous corolla lobes with usually up to only two gland-dots near the apex abaxially (Fig. 1).

#### Type.

Malaysia. Peninsular Malaysia: Terengganu, Hulu Terengganu, G. Padang, trail to summit of G. Padang, 4°51.06'N, 102°53.22'E, 1236 m alt., 20 March 2010 (fl.), *Mohd*. *Hairul et al.*, *FRI70884* (holotype KEP!).

#### Description.

A woody shrub with about 2 m high. *Indumentum* of scale or short, brown, simple or branched trichomes, with or without glands on vegetative and reproductive part. *Leaves* alternate; petiole stout, 1–2 cm long, covered with dense scale; lamina subcoriaceous, elliptic-oblong, 15–23 × 5.5–7.5 cm, base cuneate-attenuate, margin entire, apex acuminate, acumen 1–1.5 cm long, glabrous on both surfaces, except the dense, brown scale beneath; mid-rib flat above, raised below; lateral veins 21–28 pairs, closely spaced, brochidrodromous with double looping in the margin, distinct on upper surface, prominent beneath, intersecondary veins present within each pair; intercostal veins reticulate, distinct on both surfaces. *Inflorescences* axillary in the uppermost axils on lateral branches (see Notes), paniculate, ca. 12 cm long, 2 times branched, with flowers umbelliform at the ends of alternate branches, laxly to closely arranged on branchlets; peduncle and rachis 10 cm long, flexuous and winged, densely hairy; bracts lanceolate, ca. 1 mm long, glabrous on both surfaces, margin ciliate, deciduous. *Flower* 5-merous; pedicels 4–10 mm long, slender and obconically flared towards calyx base, covered with simple brown hairs, sparsely to glabrescent; calyx lobes not overlapping, spreading, covered with 2–4 brown gland-dots abaxially, glabrous on both surfaces, triangular, 1–1.2 × 1 mm, margin ciliate, with laxly spaced, pale brown hairs, apex obtuse; corolla contorted, lobes pinkish, with up to two gland-dots near apex abaxially, ovate-acuminate, ca. 3.5 × 1.5 mm, glabrous on both surfaces; stamens subsessile, anther lanceolate-mucronate, ca. 2 × 0.8 mm, glabrous, except densely covered with gland-dots near mid-rib abaxially, thecae not locellate, dehiscent by longitudinal slits; ovary globose, ca. 1 × 1 mm, glabrous, style and stigma slender, ca. 4 mm long, ovules ca. 12 in two series. *Fruits* with dense gland-dots, globose, ca. 4 × 4 mm, glabrous.

#### Distribution.

Endemic in Peninsular Malaysia, Terengganu (G. Padang).

#### Ecology.

Growing in primary lower montane forest.

#### Etymology.

The species is named after the late Dr Timothy C. Whitmore (1935–2002), a tropical botanist whose interests pertained to all aspects of tropical rain forests and who first collected this species from G. Padang.

#### Conservation status.

Least Concern (LC). This species is found only in one locality and G. Padang is under Taman Negara, which is a protected area. In addition, the habitat is an intact mossy forest where a healthy population was observed along the steep slopes ridge towards the summit plateau (Mohd. Hairul Mohd. Amin, pers. com.). Therefore, it is assessed as Least Concern (LC) according to the Malaysia Red List (Chua and Saw 2006) and IUCN Red List Categories and Criteria (IUCN 2012) and guideline version 15 (IUCN Standards and Petitions Committee 2022).

#### Additional specimen examined.

Malaysia. Peninsular Malaysia: Terengganu, Gunong [Gunung] Padang Expedition, Summit plateau G. Padang, closed 40 ft. [14 m alt.] lower montane type forest on eastern side of plateau [4°51'N, 102°52'E], 4200 ft. [1280 m alt.], 20 Sept 1969 (fr.), *T. C. Whitmore*, *FRI 12727* (KEP!).

#### Notes.

This species was initially flagged as distinct by Stone who assumed it to be similar to *Ardisiasessilis* Scheff., no doubt due to the leaf size and the venation, but to date, there is no valid name for this taxon. Although the new species shows some similarity to *A.sessilis* in the shape of the leaves (elliptic-oblong), which are in the same size range (15–25 cm long) and in the reticulation (intercostal veins ± reticulate), it differs from the latter in several morphological characteristics, such as the marginal veins absent (but double marginal veins present in *A.sessilis*), the inflorescence rachis is slender (vs. stout) and the pedicel is longer and slender (vs. short or almost sessile and thick).

There are several members of §*Stylardisia* that have large leaves and slender inflorescences rachis, but the new species most resembles *Ardisianurii* Furtado in having elliptic-oblong leaves and a brochidrodromous venation. However, the inflorescence in *A.nurii* is usually branched to three and rarely two orders, whereas in *A.whitmorei*, it is branched to two orders. In addition, the brochidrodromous venation is double looped in *A.whitmorei*, but not in *A.nurii*. The new species is also similar to *A.pterocaulis* Miq. (*A.platyclada* King & Gamble sensu Stone (1989)), also with the inflorescence rachis being slender, but *A.whitmorei* has inflorescences branched to two orders (vs. to three orders in *A.pterocaulis*), has longer leaves, 15–23 cm long (compared to the shorter leaves, 9.5–13 cm long) with a flat lamina surface (vs. bullate), the brochidrodromous lateral veins (vs. meet in prominent looped intramarginal veins) and the corolla lobes are pinkish (vs. waxy white), that are abaxially covered with only two gland-dots near the apex (vs. over the entire surface).

The material of *A.whitmorei* currently available for study is rather poor and the inflorescences are found in axils of terminal leaves, but as these inflorescences are large, multi-flowered and paniculate, as well as the flowers having the style projecting from the bud, we are confident this species is best placed within §*Stylardisia*. The material appears to be of plants that have had the terminal bud removed (they appear damaged at the apex) and we assume the inflorescences have had to appear from lower axils. Other subgenera with axillary inflorescences include §*Pimelandra* and §*Akosmos*, but the new species has none of the characters for those taxa, i.e. short axillary inflorescences or axillary and terminal inflorescences and both with no style extension prior to anthesis.

Excluding *Conamomumutriculosum* Ridl. (synonym: *Amomumutriculosum* (Ridl.) Holttum), about ten taxa are listed as endemic to G. Padang (Ummul-Nazrah et al. 2011; with more not yet named due to incomplete material, but known to be distinct from known species). The addition of the new species described here brings the total number of endemic species for G. Padang to 11, suggesting that there are very likely more taxa that may be endemic and waiting to be described.

## Supplementary Material

XML Treatment for Ardisia whitmorei
